# Estimation the direct cost of inflammatory bowel disease in Iranian patients; the one- year follow-up 

**Published:** 2019

**Authors:** Hedieh Balaii, Meysam Olfatifar, Sepideh Olianasab Narab, Asghar Arab Hosseini, Ali Seyed Salehi, Shabnam Shahrokh

**Affiliations:** 1 *Basic and Molecular Epidemiology of Gastrointestinal Disorders Research Center, Research Institute for Gastroenterology and Live Diseases, Shahid Beheshti University of Medical Sciences, Tehran, Iran*; 2 *Gastroenterology and Liver Diseases Research Center, Research Institute for Gastroenterology and Liver Diseases, Shahid Beheshti University of Medical Sciences, Tehran, Iran *

**Keywords:** Inflammatory bowel disease, Medical cost, Crohn's disease, Ulcerative colitis

## Abstract

**Aim:**

We conducted this study to estimate the direct medical cost of Iranian IBD patients.

**Background:**

In the economic evaluation setting, descriptive epidemiological studies can provide substantial information for health system policymakers in taking accountable decisions for diseases such as Inflammatory Bowel Disease (IBD).

**Methods:**

To do so, we used a self-designed checklist to collect demographic and medical cost information for IBD patients. We also tried to have a national estimation of IBD costs.

**Results:**

The mean annual medical cost of IBD was 18354.52 PPP$. Crohn's disease (CD) vs. ulcerative colitis (UC) and UC township patients vs. Tehran resident patients had higher medical costs (31160.79 PPP$; P<0.001) and (20840.23 PPP$, P<0.025). The largest medical cost spent in both IBD subtypes (CD/UC) was attributed to biological agents, especially in UC patients. We estimated that the mean annual cost of IBD in Iran for 2017 was 746315864 (95% CI: 602964172, 964685749) PPP$ (constant incidence) and 862776811 (95% CI: 697055402, 1115222835) PPP$ (increment incidence) respectively.

**Conclusion:**

Our results suggest that for management of IBD patients, policymakers should address shifting the medical costs to biological agents, the higher cost of CD, and the impact of underlying factors on the distribution of these medical costs.

## Introduction

 Inflammatory bowel disease (IBD) is a clinical condition including Crohn's disease (CD) and ulcerative colitis (UC) (1,2). The chronic, prevalent, non-fatal and disabling diseases (3) can affect other organs beyond the gastrointestinal system tract such as skin, joints, and eyes (1), and as such it can affect the patient's quality of life (4). This, subsequently, imposes a considerable economic burden that has been approved by different studies (5,6). However, along with the rapidly increasing incidence of IBD in Asia (7) and Iran (8), the economic picture of IBD in Iran has remained unclear.

In all health care systems, there is a requirement for controlling costs and accepting the need for more detailed information on the cost of diseases (9). In Iran, along with‏ the lack of accurate records on patient's resource utilization, access to accurate cost information of diseases is not possible correctly. Likewise, our knowledge of health care costs for IBD is extremely limited. On the other hand, according to severity, location, and patient treatment history, the therapeutic and surgical strategies of IBD are various and complex (10). The economic burden of IBD has been changing with the use of biologic drugs as well as diminished hospital and surgical values (11). Hence, for optimizing the treatment strategies of IBD, further research is necessary (10).

Regarding the above-mentioned points and some features of IBD such as its chronic nature, the need to treatment over the lifetime, high cost of treatment options, hospitalization, and its markedly increasing rates in Iran over the last decades (12), it is indispensable to assess the direct medical cost associated with disease to delineate the economic burden of IBD which can be different in terms of biological agents, hospitalization, surgery, and demographic factors (13,14). Accordingly, the descriptive epidemiological studies are substantial for the health system by providing important information for policymakers (15); hereupon, our study was conducted to estimate the economic costs of IBD in Tehran province during the one-year follow-up. 

## Methods

**Setting and cost analysis**

We included all cases of inflammatory bowel disease referring to Gastroenterology and Liver Disease Research Center at Shahid Beheshti University of Medical Sciences, between 2017 and 2018. We used a self-designed checklist for collecting of patient’s information, so that in each case the demographic information including sex, age, residence, education, marital status, and clinical data such as disease duration, type of disease (CD/UC), number of doctor’s visits (GP/specialist), hospitalization days, drug history, surgery, number of colonoscopies, endoscopies, and blood laboratory examinations during one year were collected. The checklists were filled out through asking questions from the patients and using their records, if necessary. The price of receiving different services was obtained from Iranian claim data. In each case, by summing the price of different provided services within one year for each person, the cost of services received for that person was calculated. We used the Purchasing Power Parity Dollar (PPP$), a popular index to inter-country comparison, for adjustment of the medical cost of IBD. Briefly, PPP adjusts the price of similar goods in a different country and does not markedly fluctuate with time (16). More details can be obtained elsewhere (17–19). 

**Statistical Analysis**

We used descriptive statistics to describe different baseline demographic and clinical information of IBD patients (CD and UC). The Pearson chi-square test and the student t-test (or its nonparametric equivalent test) were used for categorical and continues variables respectively. We also used a t-test and one-way ANOVA (or their nonparametric equivalent tests) to compare the cost of IBD (CD/UC) in terms of baseline variables. We also used the post hoc (Scheffe) test if a significant relationship was observed. Note that except for the total cost of UC and CD, which are compared with each other, we did not compare the costs of UC and CD in terms of baseline variables as in most studies (20,21) CD has had a higher cost than UC. The significance level for all of the hypothesis testing was considered at P<0.05. 

Furthermore, we tried to estimate the mean annual cost of IBD for the entire country based on our results and by considering some national sources (8,22). To this aim, we multiplied the mean annual cost per patient of IBD (obtained from our study) by their corresponding estimated number of patients (the numerator of prevalence estimate). In this way, we first extracted the population of people older than 15 years from 2016 census data reported by the statistical center of Iran (as most of the cases referring to our center, as well as those considered in the national report, were between the ages of 15 and 100 years). We then calculated the prevalence of the IBD for 2017, while taking into account its annual incidence and mortality rates. We considered the death rate at 0.19 per 100 000 (the lower bound of IBD death rate reported for Middle East (23), while we assumed that Iran had lower death rates). We first assumed that the incidence of IBD has remained constant from 2012 to 2017 and then assumed that it has increased by 0.05 per year from 2012. We used the following formula to adjust for this increment P_t+n_=P_t _(1+0.05)^ n^ where P_t _is the current estimation of incidence and Pt_+n_ denotes the subsequent estimation of incidence after n years. 

**Table 1:** Demographic characteristic of study participants

variables	UC(n=193)	CD(n=57)	P-value
Sex[n%]			0.272
Male	81(32.7)	29(11.7)	
Female	109(44.13)	28(11.34)	
Education[n%]			0.701
Primary	18(7.35)	5(2.04)	
High. School	66(26.94)	23(9.39)	
University	105(42.86)	28(11.43)	
Marital[n%]			0.758
Married	134(54.69)	38(15.51)	
Single	50(20.41)	18(7.35)	
Divorced	4(1.63)	1(0.41)	
Age[mean, CI]			
30 year	23.74(22.8-24.6)	22.78(20.3- 25.2)	0.373
>30 year	43.42(41.3- 45.5)	44.4(39.9- 48.8)	0.694
Residence			0.772
Tehran	46(34.07)	33(24.44)	
Township	34(25.19)	22(16.30)	

**Table 2:** The medical costs of IBD (UC/CD) patients including mean and CI95 % (costs are based on PPPS$)

variables	UC(n=190)	P-value	CD(n=57)	P-value
Sex		0.514		0.166
Male	16127.94(10464.2-21791.6)		40866.65(13711.6- 68021.6)	
Female	13696.01(8910.8-18481.1)		21108.29(12727.5-29489.1)	
Education		0.599		0.068
Primary	15155.08(7526.3-22783.7)		82481.12(-29589.8-194552.1)	
High. School	17108.62(11132.1-23085.1)		21233.93(14363.1-28104.7)	
University	13063.1(7838.8-18287.4)		30489.33(10944.3-50034.2)	
Marital		0.002		0.818
Married	10651.42(8221.3-13081.5)		30017.36(15556.6-44478.04)	
Single	25262.65(13688.4-36836.8)		35193.49(2577.06-67809.9)	
Divorced	12847.33(-9532.1-35226.7)		2022.46(.)	
Age		0.001		0.790
≤ 30 year	26918.72(15374.33-38463.1)		37069.04(4389.2-69748.8)	
>30 year	9898.16(7481.8-12314.4)		32547.05(13785.4-51308.6)	
Residence		0.025		0.995
Tehran	10452.27(5820.4-15084.1)		31254.79(12368.6-50140.9)	
Township	20840.23(10184.2-31496.2)		31173.01(5581.2-56764.7)	
Total	14572.36(11002.0-18142.7)	-	31160.79(17178.6-45142.9)	0.001

Finally, due to the inherent skewness of cost data, we reported the bootstrapping Bias Corrected and Accelerated (BCa) confidence interval (CI) with 10000 replications for the mean annual cost of IBD.

**Table 3:** The mean medical costs spent during one year of follow-up on all type of cares for UC and CD patients (costs are based on PPP$)

Items	UC(n=193)	CD(n=57)
Mesalazine	355.64	195.60
Sulfasalazine	34.16	19.68
Tablet Asacol 800	1084.15	759.35
Tablet Asacol 400	277.24	130.54
Pentasa	170.12	570.15
Azathioprine	61.65	72.26
Prednisolone	16.73	13.17
Cortenema	54.46	9.17
Ferrous-Sulfate	0.12	-
Folic Acid	2.14	1.92
Calcium	0.49	0.63
Vitamin D	3.55	4.36
Infliximab	2957.62	8894.58
Asacol supp.	4584.91	553.19
Cinnora	15805.47	-
Enema asacol plus Asacol	9179.33	5471.12
Enema Asacol	7349.11	9118.54
Humira	36474.16	43769
Mesalazine supp.	91.18	-
Pentasa supp.	2772.03	2772.03
Visit by a General practitioner	2.19	5.87
Visit by other Specialist	29.93	16.21
Visit by gastroenterologist	213.79	668.69
Colonoscopy	400.02	383.94
Blood lab exam	149.09	220.41
CMV	66.47	89.36
CDIFF	59.08	92.81
Calprotectin	3.05	2.58
Endoscopy	79.79	149.31
Hospitalization	6590.88	14712.75

## Results

**Primary analysis**

In total, 259 IBD patients were included in our study with nine cases excluded owing to their incomplete information (we did not include cases with incomplete demographic information). Out of 250 remained IBD patients 193 (77.2) and 57 (22.8) were UC and CD, respectively. Of these, 110 (44.53) cases were male and 137 (55.47) were female. Most of the UC cases were female 109 (44.13), had university education 105 (42.86), and had been married 134 (54.69). On the other hand, most of the CD cases were male 29 (11.7) with university education 28 (11.43) and married 38 (15.51). The mean age of UC and CD cases was 37.82 and 36.02 respectively. We did not observe any significant relationship between IBD subtypes (UC/CD) in terms of baseline variables (Table 1).

**Cost of illness** **analysis **

Considering the importance of cost evaluation in the health care system and to outline the economic burden of IBD, we estimated the cost of different medical services provided in Gastroenterology and Liver Disease Research Center for IBD patients. We only compared the total medical cost of UC/CD with each other (intergroup comparability) and for another comparison only checked the intragroup comparability of UC/CD for each of the baseline variables. The results showed that the total annual cost and the average annual cost for IBD patients were 4588631 PPP$ and 18354.52 PPP$ respectively. On the other hand, the mean medical costs for CD (31160.79 PPP$) were higher than for UC (14572.36 PPP$; P<0.001) (Table 2). We observed that the average medical cost in UC patients with <30 age year (26918.72 PPP$) was higher than in patients with >30 age (9898.16 PPP$, P<0.001) (Table). Likewise, Scheffe post hoc test showed that the mean medical cost for married UC patients was different from that of single patients (P=0.002). In other cases, we did not observe any significant relationship (Table 2). Also, for UC patients the mean cost of township patients (20840.23 PPP$) was higher than for resident patients of Tehran (10452.27, P=.025), but in CD patients we did not observe any significant relationship (P=.994) (Table 2). These results can indicate that the magnitude and direction of medical costs are different in patients with UC and CD.

Considering the importance of recognizing the contribution of each service to resource utilization, we reported the mean cost of provided services. The most spent medical costs in UC were related to Humira (36474.16 PPP$), Cinnora (15805.47 PPP$), Enema Asacol plus Asacol suppository (9179.33 PPP$), Enema Asacol (7349.11 PPP$), hospitalization (6590.88 PPP$) and Infliximab (2957.62 PPP$). Likewise, the largest spent medical costs in those with CD were related to Humira (43769 PPP$), hospitalization (14712.75 PPP$), Enema Asacol (9118.54 PPP$), Infliximab (8894.58 PPP$), Enema Asacol plus Asacol suppository (5471.12 PPP$) and Pentasa suppository. (2772.03PPP$) (Table3). These results can approve the shifting of IBD cost to biologic drugs, especially in UC patients.

**Cost of illness for the country**

We observed that the mean annual cost per patient of IBD was 18354.52 (95% CI: 14829, 23725) PPP$. On the other hand, the prevalence of IBD in 2012 was 40.67 per 100000. If we assume a constant incidence from 2012 to 2017, the prevalence and subsequently the mean annual cost of IBD in 2017 were 66.95 per 100000 and 746315864 (95% CI: 602964172, 964685749) PPP$. The prevalence and mean annual cost of IBD for another scenario were 77.39 per 100000 and 862776811 (95% CI: 697055402, 1115222835) PPP$ respectively.

## Discussion

In general, our results can assist policymakers to make better decisions for the management of inflammatory bowel diseases considering the higher cost of IBD in Crohn's disease, shifts in medical cost to biological agents, and different role of demographic and socioeconomic factors on the distribution of these medical costs. We observed that the medical cost in patients with CD was higher than that in UC patients both on average (31160.79 PPP$ vs.14572.36 PPP$) and for most medical services. Our results imply that the economic burden of IBD has shifted from surgical and hospitalization values to biologic drugs, where for both IBD subtypes (UCCD), the highest medical cost, with a major difference, was attributed to Humira (36474.16 PPP$ and 43769 PPP$). Meanwhile, our results showed that demographic factors can play a different role in the distribution of IBD medical costs. 

As an alternative result, we estimated the mean annual cost of IBD for the country. We assumed that there was the same population heterogeneity between our study and the national report. However, these results may have some degrees of overestimation and/or underestimation. 

Consistent with other pieces of evidence, the CD patient's usage (benefit) of medical services is 2-4 times (CD_cost:_ UC_cost, _31160.79/14572.36=2.13) greater than that of UC patients as opposed to their prevalence (UC: CD, 193/53=3.6)(20,24). It seems that this unbalanced ratio of prevalence is dominant throughout Iran, at least for 2012 when the prevalence of UC and CD was reported as 35.52, and 5.03 per 100,000 people respectively(8). On the other hand, these observed imbalance ratios/rates are relatively different in some developed countries and much lower; in 2009 for adults the UC: CD_ratio_ was 263/241=1.091 and in children, and the respective ratio was 34/58=0.58(25). Three other studies reported relatively similar results (26–28). The reasons for this unbalanced ratio can be attributed to the different factors such as the natural history of IBD as well as some other etiological and pathological factors that we cannot explain.

Along with our study, most of studies have attributed the high resource utilization to biological agents (29) which can approve the shifting of the medical cost to biological agents. These findings and markedly increasing (12) incidence of inflammatory bowel disease in Iran can induce the need for adoption of more appropriate strategies not only for the emergence of epidemiology (8) of IBD in Iran but also for managing the high costs associated with biological agents. It is because these costs are staggering and are hard to afford for both the health system and patients (29). 

Likewise, another piece of evidence revealed that the distribution of medical costs can be affected by different demographic and socioeconomic factors. So that, one study showed that patients with higher-need to medical services and subsequently those with higher-cost are more likely to be in a lower-income level, obese, and have comorbidity (30). Consideration of this heterogenous distribution can be very important as identifying people with high resource utilization is the first step in health system management policies (30). However, absence of a significant relationship for CD patients in terms of investigated variables can be attributed to the small sample size in this group (Tables 1, 2), which is not very unexpected given observation of a similar situation in other studies (29). 

Meanwhile, patients’ resource utilization and subsequently the optimal treatment of IBD which requires specialized health care can be affected by patients traveling long distances to obtain this specialized health care (31). As mentioned earlier, the incidence rate of IBD is growing in Iran, and ac 1) more than %60 of gastroenterologists live in Tehran and in ten major provinces of the country; 2) most of the treatment services are located in tertiary care centers and many of patients refer to Tehran to obtain an optimal treatment (8). Hence, we can consider the adverse effect of the long waiting list on patients and resource utilization (32) where probably the same also holds for our participants, considering the higher medical cost for UC township patients vs. Tehran resident patients. Hence, the implementation of Population Health Management strategies can be very helpful and cost-effectiveness as confirmed and discussed by multiple studies (33,34).

In our study, the non-medical direct-cost of provincial patients referring to Tehran province such as transportation and accommodation, food, and telephone usage cost were not estimated. Consideration of these costs can significantly change the medical cost of these patients and can be used in decision-making processes. Note that we did not consider indirect medical costs either (35).

Taken together, our results suggest that to better manage inflammatory bowel disease and provide optimal treatment for these patients, policymaker's and clinicians should consider the important role of biological agents, heterogeneous distribution of costs among patients (in terms of baseline variables) and higher medical as well as additional non-medical costs in township patients. In light of this, further research is warranted at national and provincial levels to gain a comprehensive perspective.
